# Asymmetric cell division of stem and progenitor cells during homeostasis and cancer

**DOI:** 10.1007/s00018-013-1386-1

**Published:** 2013-06-15

**Authors:** Sandra Gómez-López, Robin G. Lerner, Claudia Petritsch

**Affiliations:** 1 Department of Neurological Surgery, University of California, San Francisco, San Francisco, CA USA; 2 Brain Tumor Research Center, University of California, San Francisco, San Francisco, CA USA; 3 Helen Diller Comprehensive Cancer Center, University of California, San Francisco, San Francisco, CA USA; 4 Eli and Edythe Broad Center of Regeneration Medicine and Stem Cell Research, University of California, San Francisco, San Francisco, CA USA

**Keywords:** Asymmetric cell division, Polarity, Neural stem cell, Oligodendrocyte precursor cell, Glioma, Tumor-propagating cell, Cancer stem cell

## Abstract

Stem and progenitor cells are characterized by their ability to self-renew and produce differentiated progeny. A fine balance between these processes is achieved through controlled asymmetric divisions and is necessary to generate cellular diversity during development and to maintain adult tissue homeostasis. Disruption of this balance may result in premature depletion of the stem/progenitor cell pool, or abnormal growth. In many tissues, including the brain, dysregulated asymmetric divisions are associated with cancer. Whether there is a causal relationship between asymmetric cell division defects and cancer initiation is as yet not known. Here, we review the cellular and molecular mechanisms that regulate asymmetric cell divisions in the neural lineage and discuss the potential connections between this regulatory machinery and cancer.

## Introduction

Asymmetric cell division (ACD) is a fundamental process whereby the asymmetric inheritance of cellular components (e.g. proteins, RNAs) during mitosis defines distinct fates for each daughter cell. This evolutionarily conserved division mode is used by stem and progenitor cells in different tissues. In a typical outcome of an asymmetric division, the stem or progenitor cell generates a copy of itself, which retains self-renewal ability and differentiation potential, and one daughter that enters the path of differentiation. Thus, by balancing self-renewal with differentiation, asymmetric divisions maintain the stem and progenitor cell pool while allowing the generation of diverse functional cells.

Much of what we know about the regulation of ACD is gleaned from studies of the stem-like cells of the central nervous system of *Drosophila*, the neuroblasts (NBs). These studies in *Drosophila* have shown that disruption of ACD leads to abnormal proliferation and genomic instability, indicating that ACD may operate as a tumor suppressor mechanism during normal development [1]. Investigations of the mammalian central nervous system have more recently revealed that neural stem cells (NSCs) and oligodendrocyte precursor cells (OPCs) undergo ACD [2, 3]. Moreover, decreased ACD frequency has been found in cancers with a stem and progenitor foundation, such as leukemia [4], brain tumors [3], and mammary carcinomas [5]. Intriguingly, however, a subset of highly tumorigenic cancer cells with stem cell properties, the cancer stem cells (CSCs), retain their ability to divide asymmetrically in established brain tumors [6], suggesting that ACD may play an important role in tumor maintenance. We will therefore first describe how ACD is established in *Drosophila* NBs and subsequently discuss the extent to which these mechanisms appear to be conserved in the mammalian neural lineage. In the final part of this review, we will discuss the emerging roles of ACD regulators in controlling cellular features observed during the initiation and progression of human cancers.

## Asymmetric divisions of *Drosophila melanogaster* neuroblasts

*Drosophila* NBs are the most thoroughly studied model system of ACD, where basic principles of polarity, spindle orientation, and cell-fate determination have been revealed [7]. Embryonic NBs undergo several rounds of asymmetric divisions, during which determinants of differentiating fate concentrate at the basal cell cortex before mitosis and segregate unequally during cytokinesis, to generate each time another NB and a more restricted progenitor called ganglion mother cell (GMC). At early stages of larval development, and after a period of quiescence, NBs re-enter the cell cycle and continue to divide asymmetrically to produce GMCs, either directly (type I NBs) or via intermediate progenitors (type II NBs) [8–10].

### Establishing polarity

Embryonic NBs delaminate from a polarized neuroectoderm and inherit apically positioned Bazooka (Baz or Par3) protein. Baz serves as an apical polarity cue and during late interphase/early prophase, assembles a polarity complex [11]. Baz binds and activates the Rho GTPase family Cdc42 [12], which in turn recruits atypical protein kinase C (aPKC) and the aPKC inhibitory subunit Par6 [12, 13]. In prophase, the apical complex also binds to the adaptor protein Inscuteable (Insc) [14] and thereby initiates the assembly of a second complex consisting of partner of Insc (Pins) [11] and the heterotrimeric G protein coupled subunits Gαi and Gβγ. Pins-dependent heterotrimer formation of Gαi/βγ activates G protein signaling in a transmembrane receptor-independent manner [15] and in the absence of nucleotide exchange [16].

**Fig. 1:**
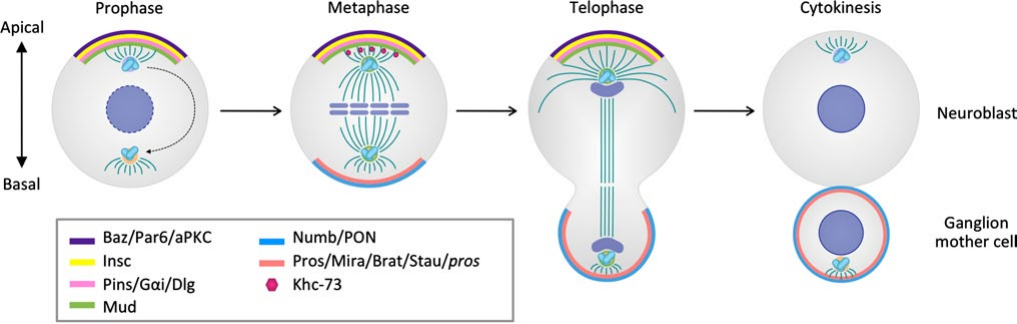
Asymmetric division in *Drosophila* neuroblasts. Polarized localization of apical complexes is established during prophase. During metaphase and telophase, the spindle is anchored and orientated relative to the axis of apico-basal polarity. Cell-fate determinants are asymmetrically segregated into self-renewing neuroblasts or differentiating ganglion mother cells

In metaphase, the mitotic kinase Aurora A (AurA) phosphorylates Par6, which in conjunction with Baz/Cdc42 binding leads to aPKC activation [14, 17, 18]. Protein phosphatase 2A (PP2A) restricts active aPKC to the apical cortex in larval NBs [19, 20] and dephosphorylates Baz and Par6 in embryonic NBs [17, 21]. Thus, NB polarity is established through the dynamic physical association of scaffold proteins, which coordinate GTPase, kinase, and phosphatase activities. The activation of G protein signaling through Pins occurs cell intrinsically and not only stabilizes apical polarity but also positions the nascent mitotic spindle along the apico-basal axis and determines its size asymmetry (Fig. 1).

### Orienting the mitotic spindle

Proper apico-basal spindle positioning depends on a dynamic cross-talk between polarity and spindle-orientating complexes at the cortex with centrosomes and astral microtubules. Shortly after cytokinesis is completed, NBs prepare for the next round of division by localizing one centrosome to the vicinity of the apical pole. The apical centrosome forms astral microtubules and anchors in a Pins-dependent manner. After it duplicates, the mother centrosome moves away to the basal pole [22]. In actively cycling larval NBs, the apical centrosome provides polarity cues that precede those provided by the apical polarity complex and it functions as a spatial memory for proper spindle axis formation in subsequent rounds of divisions [22–24].

The coiled-coiled domain protein mushroom body-defective (Mud) binds to the centrosome and links astral microtubules to the apical complex by interacting with Pins [25–27]. The microtubule-cortex contact is additionally controlled by the MAGUK Disc large (Dlg), which physically interacts with Pins at the cortex and with kinesin Khc-73 at the microtubule plus ends [28, 29]. Pins/Mud interactions stabilize the mitotic spindle and Pins/Gαi/Dlg/Khc-73 interactions establish a positive feedback loop, and thereby maintain apical cortical polarity as well as the correct spindle orientation.

The temporal coordination of spindle positioning is less well understood and it is known to require the Polo and AurA kinases [30–32]. Polo kinase is part of the spindle assembly checkpoint that ensures that microtubules are properly connected to kinetochores and that chromosomes segregate correctly. Polo therefore potentially links the temporal control of ACD with the global temporal control of mitosis and proper ploidy.

### Cell-fate decision control

The Baz/Par6/aPKC complex directs the basal distribution of the cell-fate determinant Numb, an evolutionary conserved protein primarily known for its ability to inhibit the Notch signaling pathway, and the adaptor protein Miranda (Mira). This process depends on an intact actin cytoskeleton [33] and two myosin motors, MyoII and MyoVI, which operate downstream of the apical complex [34].

In interphase, aPKC and Par6 form a complex with the WD40 protein lethal giant larvae (Lgl). Mitotic phosphorylation of Par6 by AurA stimulates aPKC to phosphorylate Lgl, which is then released from aPKC, allowing Baz to enter the Par complex [18, 35]. Assembly of Baz/Par6/aPKC changes aPKC substrate specificity, allowing it to phosphorylate Numb and releasing it from the apical cortex [18, 36]. Phosphorylated Numb then localizes to the basal side of the cell [36] assisted by the adaptor protein Partner of Numb (PON) [37], whose activity is largely dependent on its phosphorylation by Polo kinase [30]. Once in the GMC, Numb prevents self-renewal, predominantly by antagonizing Notch signaling [31].

Similar to Numb, Mira polarization has been proposed to occur as a consequence of direct aPKC phosphorylation [18, 38]. However, in *aurA* mutant NBs, which display delocalized aPKC, Mira distribution is unaltered [31, 32], implying the existence of alternative mechanisms acting upstream of or in parallel to aPKC. Mira binds to and directs the asymmetric localization of distinct cell-fate determinants, including the transcriptional regulator Prospero (Pros) [39, 40], the double-stranded (ds) RNA-binding protein Staufen (Stau) [41, 42] and the NHL-domain protein Brain tumor (Brat) [43, 44]. Stau binds to *pros* mRNA, through its 3’ UTR, and localizes it to the GMC [45, 46], which itself does not transcribe the *pros* gene [46]. In the GMC, Prospero activates a neurogenic program [47, 48] and represses expression of genes associated with stem cell fate and cell-cycle progression [49]. Brat, on the other hand, promotes cell-cycle exit, represses NB fate, and inhibits cell growth [43, 44, 50], at least in part, by inhibiting the expression of the transcription factor dMyc at a post-transcriptional level [43].

As a result of unequal segregation of basal cell-fate determinants, the apical NB daughter continues to proliferate, while the basal GMC daughter is committed to differentiate (Fig. 1). The specification of NB and GMC fates is irreversible and no cases of spontaneous de-differentiation of a GMC into a NB have been observed.

## Asymmetric cell division in mammalian neural lineages

The production of the different cell types in the mammalian central nervous system (CNS) occurs in temporally defined, though overlapping, developmental phases. Generally, neurons arise first, followed by astrocytes and oligodendrocytes. This process is largely determined by cell-intrinsic changes that alter the differentiation potential of CNS progenitors as development proceeds [51, 52].

### Polarized progenitors and division mode during embryonic neurogenesis

Similar to *Drosophila* NBs, mammalian embryonic neural stem cells, or radial glia (RG) cells, arise from neuroepithelial cells, with which they share pronounced apico-basal polarization. RG cells divide in the ventricular zone (VZ) and they contact the ventricular and pial surfaces of the neural tube through an apical process and a long basal fiber, respectively. At its apical endfoot, each RG cell forms close contacts with its neighboring cells via adherens junctions (AJs) [53, 54].

**Fig. 2:**
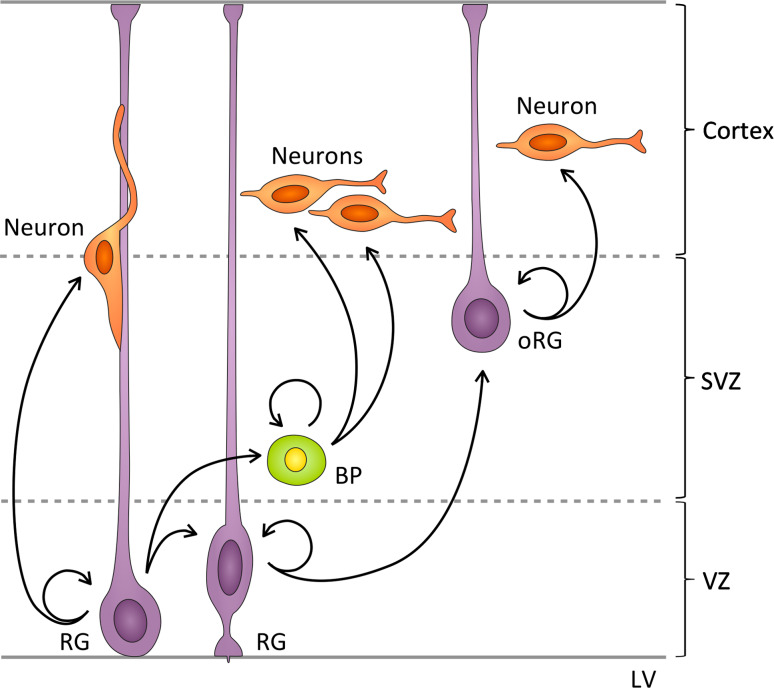
Patterns of cell division during mammalian embryonic neurogenesis. During the peak period of murine neurogenesis radial glia (*RG*) cells divide in the ventricular zone (*VZ*) mainly asymmetrically, generating one RG cell and one neuron, or one RG cell and one basal progenitor (*BP*), which migrates to the subventricular zone (*SVZ*). Asymmetric RG divisions also produce outer RG (*oRG*) cells, which lose their ventricular attachment and translocate to the SVZ, where they divide asymmetrically to produce neurons

During the peak of neurogenesis, RG cells execute mainly asymmetric self-renewing divisions to produce either neurons or basal progenitors (BPs) [55]. The newly born neurons and BPs lose both ventricular and pial attachments and migrate basally [2, 56]. BPs then divide symmetrically in the subventricular zone (SVZ) to give rise to either two BPs, or, as in most cases, to two neurons that migrate further basally [2, 56] (Fig. 2). Another type of neurogenic progenitor with a short basal fiber has been reported to divide adjacent to RG cells, which has led to the proposal that multiple types of neural progenitors co-exist in the VZ [57].

Recently, a new class of RG residing in the outer SVZ of the human, ferret [58, 59], and mouse embryonic brain [60] has been identified. Murine outer RG (oRG) arise from asymmetrically dividing RG cells and, in contrast to BPs and neurons [60], oRG inherit the basal fiber, but lose their apical attachment [61] and migrate to the SVZ, where they undergo asymmetric self-renewing divisions to produce neurons [60] (Fig. 2).

#### The Par complex and adherens junctions

The molecular mechanisms that establish polarity in RG and oRG cells are only partially delineated. The mammalian homologs of the polarity regulators Par3, Par6, and aPKC [62], as well as of Cdc42 [63], localize to RG apical endfeet and appear to serve two main functions: First, they maintain apical AJs integrity, thereby preserving RG polarity and VZ organization, and secondly, they participate in the control of RG cell fate.

Apical assembly of the Par3/Par6/aPKC complex requires Cdc42 function. Targeted deletion of Cdc42 disrupts apical localization of Par6 and aPKC [64] and causes loss of AJs [63]. Similarly, conditional depletion of aPKCλ in RG leads to loss of AJs, disruption of the VZ, detachment of apical processes from the ventricular surface, and centrosome mislocalization. Despite these polarity defects, neurogenesis occurs normally [65]. This may either indicate that maintenance of AJs integrity and associated polarity are dispensable for cell fate determination, or alternatively, that loss of aPKCλ may not completely disrupt the intrinsic polarity of RG, perhaps due to functional redundancy with other aPKC isoforms.

RG cells AJs are formed by three different domains, which are sometimes bisected in an asymmetric fashion by the mitotic spindle during RG divisions [66]. A basal β-catenin/E-cadherin-positive region and a central ZO1^+^ domain segregate to both daughter cells in the majority of the divisions. In contrast, an apical region enriched for Par3 and aPKC, together with the most apical part of the plasma membrane, which shows localized expression of Prominin-1, have been found to be consistently distributed to only one daughter cell in neurogenic divisions [66, 67].

#### Prominin-1 in the VZ

Prominin-1 (Prom1) is a multispan transmembrane glycoprotein that is specifically sorted to microvilli and plasma membrane protrusions and localizes to membrane microdomains that are released into the extracellular space [68]. The human homolog of Prom1, CD133, is a cell-surface marker for distinct, malignant populations of CSCs, and has been shown to segregate asymmetrically in a subpopulation of brain CSCs, which we will discuss further below [6]. CD133/Prom1 also segregates asymmetrically in hematopoietic stem and progenitor cells, suggesting a broader and common role in somatic stem and progenitor cells [69]. So far, described phenotypes for genetic depletion of *Prom1* in mice are restricted to retinal degeneration [70]. Therefore, the significance of the asymmetric distribution of Prom1-containing microdomains in RG and non-neural stem cells remains unclear.

#### Par3 and Numb

Gain- and loss-of-function studies are conclusive in showing that unequal segregation of Par3/aPKC is responsible for differential cell-fate determination. Forced expression of *Par3* increases symmetric RG divisions at the expense of BP generation. In contrast, conditional *Par3* depletion increases neurogenesis [71]. The effects of Par3 on RG cell fate depend on Numb and its close relative Numb-like (Numbl). Par3 seems to directly interact with Numb and thereby to modulate its antagonistic effect on Notch signaling [71]. Since Notch activity is involved in the maintenance of RG identity [72], this provides a mechanism by which Par3 promotes RG fate. Interestingly, Numb and Numbl have also been implicated in the maintenance of RG AJs and polarity [73]. Thus, Par3 and Numb/Numbl maintain polarity and balance self-renewal and differentiation of RG cells.

#### Par3-independent asymmetry

Consistent with the lack of apical process, human oRG fail to express the apical regulators Par3 and aPKC [59], yet they retain expression of the RG markers Sox2 and Pax6 and continue to divide asymmetrically [58, 59]. This suggests that a currently undefined, Par3-independent, mechanism of polarity and cell fate control exists in oRG cells. An interesting possibility is that this regulatory machinery is dependent on the basal fiber. In support of this, analyses of the contribution of the apical domain and basal RG process to cell-fate determination on murine slice cultures have shown that inheritance of the basal process alone is associated with acquisition of either RG or oRG fate, whereas inheritance of the apical domain in addition to the basal process is required to retain RG identity [61].

Given that the asymmetric inheritance of both the basal process and Par3/aPKC at the apical domain depend on a slight tilting of the mitotic spindle, these observations highlight how crucial the positioning of the mitotic spindle in relation to the apico-basal axis may be for cell-fate determination.

### Spindle orientation and centrosome asymmetry

During the peak period of neurogenesis, the majority of RG cells undergo vertical divisions, with the mitotic spindles aligned within 0° and 30° of the ventricular surface [74, 75]. A smaller fraction of the cells have an oblique spindle orientation (between 30° and 60°), and only rarely, cells divide with a horizontal cleavage plane [74, 76].

#### Pins/LGN and Insc

Similar to *Drosophila* NBs, Pins and Insc homologues play a role in orienting the spindle in RG cells, although some functional differences appear to exist. LGN, an orthologue of Pins, is highly expressed in the VZ and localizes to the lateral membrane of dividing RG cells [77]. Genetic ablation [74] or siRNA-mediated knockdown [61] of *LGN* lead to increased oblique RG divisions at the expense of vertical divisions. During oblique divisions, the entire apical endfoot with the Par polarity complex is inherited by only one cell, while the other cell remains Par3-negative. This results in abnormal production of oRG cells at the expense of differentiated progeny [61].

Interestingly, Insc seems to oppose LGN function in RG cells [74]. The two proteins do not overlap in their subcellular localization, but rather Insc localizes to an apical crescent in RG cells at prometaphase and to the midzone in anaphase. Forced expression of *Insc* in the developing mouse cortex increases the frequency of horizontal and oblique divisions [74, 75]. Conversely, conditional inactivation of *Insc* causes an increased number of vertical divisions [75]. The number of RG cells in the VZ is not affected by changes in Insc expression. However, upon Insc overexpression the overall number of proliferative cells, Pax6^+^ progenitors in the outer layers, as well as that of BPs and neurons increases [74, 75]. Postiglione and colleagues concluded that increased oblique divisions led to increased numbers of BPs directly. Yet, an alternative explanation is that increased oblique divisions may generate more oRG, which in turn produce BPs and neurons [78]. Regardless of the mechanism, these observations are difficult to reconcile with a conserved function of Insc as a cellular linker of the Par complex and LGN/Gαι.

In RG cells, the centrosome localizes to the tip of the apical endfoot and, after duplication, the nascent centrosome predominantly segregates to the differentiating progeny [79]. Thus, although showing stereotypical asymmetric segregation, the pattern of inheritance of nascent and maternal centrosome in RG is opposite to that in *Drosophila* NBs.

#### Treacle/Plk1

The phosphoprotein Treacle has been found to associate with the centrosome and to be critical for correct spindle positioning and mitotic progression. Treacle function appears to depend on its physical interaction with Polo-kinase 1 (Plk1), a mammalian Polo homolog. In mice hemizygous for *Treacle*, localization of Plk1 to the centrosome is disrupted. Either *Treacle* deficiency or pharmacological inhibition of Plk1 prolong mitoses and perturb cleavage planes of RG divisions [80]. This suggests that the Treacle/Plk1 complex acts as a checkpoint that RG cells pass once the mitotic spindle is properly positioned.

In summary, some general ACD principles appear to be conserved between NBs and RG, such as the crucial role of cell intrinsic cues, rather than external signals, in determining the orientation of cell divisions [81]. Yet, organism-specific differences exist in the regulation of cleavage plane and centrosome asymmetry.

### Cell fate determination

Some of the mouse homologs of the cell fate determinants that regulate ACD in *Drosophila* NBs have been reported to preferentially localize to either the basal fiber or apical domain of RG cells.

#### Stau2, Prox1, and Trim32

Staufen2 (Stau2), the mouse homolog of Staufen, is apically localized in RG cells [82, 83]. In vitro cell pair assays with cortical progenitors indicate that it preferentially segregates to the daughter that acquires a BP fate [83]. Stau2 is part of a ribonucleoprotein complex that includes the RNA-binding proteins Pumilio2 (Pum2) and DEAD box polypeptide 1 (Ddx1), as well as cargo mRNAs such as the mammalian homologs of *prospero* and *brat*, *Prospero*-*related homeobox 1* (*Prox1*) [82] and *Tripartite*-*motif containing 32* (*Trim32*) [83], respectively. Depletion of *Stau2*, *Ddx1*, or *Pum2* by shRNA results in premature differentiation of RG cells into neurons [82, 83], indicating that the complex is essential for progenitor maintenance. Moreover, *Stau2* knockdown was shown to cause mislocalization and increased levels of *Prox1* mRNA [82], as well as a more diffuse and symmetric distribution of *Trim32* mRNA [83]. Since Prox1 is known to mediate cell-cycle exit and neurogenesis in the neural retina [84] and Trim32 induces neurogenesis [85], these data suggest that, similar to its *Drosophila* homolog, Stau2 binds and localizes neurogenic determinants away from the self-renewing daughter.

Trim32 is an E3-ubiquitin ligase and has been suggested to promote neuronal differentiation by activating specific microRNAs, such as *Let*-*7* [85], and by enhancing the transcriptional activity of retinoic acid receptor-α [86]. Additionally, Trim32 binds to the transcription factor c-Myc and targets it for degradation, thus coupling cell-fate specification with cell-cycle exit [85]. Trim32 has also been shown to interact with membrane aPKCξ [87]. It has been proposed that this interaction prevents its nuclear translocation and consequently c-Myc degradation in neural progenitors, allowing maintenance of an undifferentiated and proliferative state.

#### Asymmetry and cell cycle

Cell-fate determination in the neural lineage is tightly linked to cell-cycle length. Indeed, lengthening G1 has been shown to be necessary and sufficient to switch neural progenitors into neurogenesis [88]. In cyclin-dependent kinase 2 and 4 (Cdk2/4) double-knockout mice, neural progenitors exhibit G1 phase lengthening, which correlates with increased spontaneous differentiation in vitro and loss of BPs and increased neuronal production in vivo [89]. Interestingly, recent work indicates that asymmetrically dividing RG cells unequally segregate Cyclin D2, a positive regulator of G1 progression [90]. With the onset of neurogenesis, both Cyclin D2 protein and mRNA become enriched at the basal endfeet of RG cells [90, 91]. This biased distribution has been proposed to result from active transport and local translation of *Cyclin D2* (*Ccnd2*) mRNA [90]. Upon asymmetric RG cell division, Cyclin D2 preferentially segregates to the nucleus of the self-renewing daughter [90], where it plays an active role in promoting RG cell fate, as suggested by functional studies [90, 92]. Together, these observations not only propose a mechanistic integration between cell-cycle regulation and cell-fate determination but also provide support to the view that inheritance of the basal process may be key for RG cell-fate specification.

### Cellular divisions in the adult neurogenic niche

**Fig. 3:**
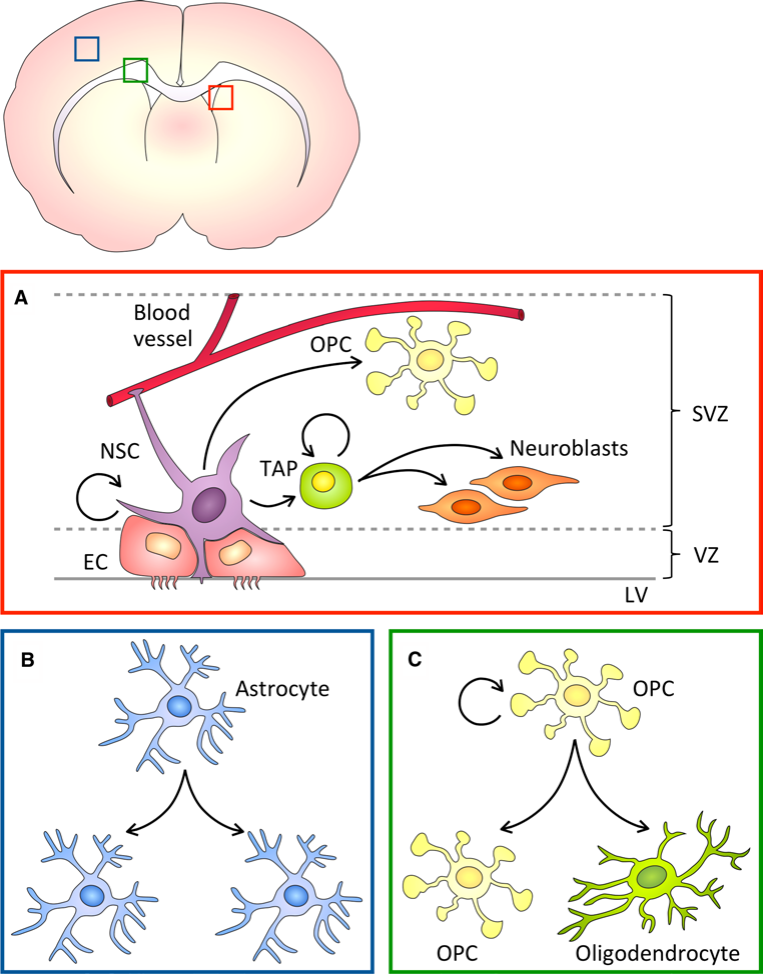
Progenitor divisions in the postnatal and adult brain. **a** Neural stem cells (*NSCs*) in the adult ventricular (*V*)-subventricular zone (*SVZ*) neurogenic niche extend contacts to blood vessels and the lateral ventricle (*LV*) and are surrounded by ependymal cells (*ECs*). NSC divisions produce transit amplifying progenitors (*TAPs*) and oligodendrocyte precursor cells (*OPCs*), which in turn divide to generate postmitotic progeny. **b** Postnatal cortical astrocytes undergo symmetric proliferative divisions. **c** In the white matter, adult OPCs divide either symmetrically or asymmetrically to self-renew and produce differentiated oligodendrocytes

By early postnatal life, following the completion of the neurogenic period, RG cells retract their processes and differentiate into astrocytes and ependymal cells [93, 94]. Yet, in the rodent adult brain, two main neurogenic sites remain: the SVZ of the lateral wall of the lateral ventricle [95, 96] and the subgranular zone of the dentate gyrus of the hippocampus [97, 98]. In these regions, a subpopulation of RG-derived cells, which express glial fibrillary acidic protein (GFAP) and Nestin, act as neural stem cells (NSCs) [99, 100]. Adult SVZ NSCs, frequently referred to as type B cells, divide to generate transit-amplifying progenitors (TAPs) [101] and oligodendrocyte precursor cells (OPCs) [102]. TAPs and OPCs in turn produce neuroblasts, also called type A cells [99], and oligodendrocytes [102], respectively (Fig. 3a).

Similar to RG cells, adult SVZ NSCs are polarized. They extend a long basal process that contacts a blood vessel, and a small ciliated apical domain that touches the lateral ventricle and forms AJs with the ependymal layer [103, 104], the integrity of which depends on Numb activity [105]. Due to the morphological similarities, it could be reasonably assumed that molecular mechanisms of polarity generation and maintenance are shared between RG and adult NSCs. However, this remains to be investigated.

Ex vivo analyses have identified a proliferative or “activated” GFAP^+^ population in the adult SVZ that distinguishes itself from a quiescent GFAP^+^ NSC population by the expression of epidermal growth factor receptor (EGFR) [106]. A recent study has demonstrated that the majority of NSCs in situ are actually quiescent and only 8.6 % of GFAP^+^ cells in the adult SVZ actively proliferate [107]. Given the high level of quiescence, it is not surprising that the in vivo division pattern of adult NSCs has not been determined yet. In situ labeling studies, however, suggest that following NSC division, TAPs undergo three amplifying divisions before giving rise to neuroblasts [107]. These data are in line with earlier in vitro time-lapse imaging studies of SVZ-derived progenitors that proposed that TAPs predominantly divide symmetrically for several rounds to expand the TAP cell pool and to generate neuroblasts [108]. This suggests that isolated adult SVZ neural progenitors preserve the cell-division pattern that they display in vivo, arguing for cell-autonomous regulation of lineage progression. Interestingly, in their in vitro imaging analyses, Costa and colleagues detected GFAP^+^ cells that divide to self-renew and to give rise to TAPs, suggesting that adult NSCs indeed undergo ACD [108].

The adult ventricular-subventricular neurogenic niche encompasses the ventricle with cerebrospinal fluid, ependymal cells, NSC progeny, and capillaries [104, 109]. The observation that NSCs contact those components raises the attractive possibility that extrinsic cues such as self-renewal factors emitted by capillaries [110, 111] and growth factors in the cerebrospinal fluid [112] may promote NSC proliferation. Interestingly, in vitro studies have shown that a fraction of adult NSCs asymmetrically distributes Notch and EGFR between daughter cells, thus producing progeny with differential response to extrinsic cues. In neurosphere cultures, high Notch activity and EGFR expression levels correlate with high self-renewal capacity [110]. Asymmetric EGFR levels between sibling cells are regulated at both transcriptional and post-translational levels, by different yet complementary mechanisms. First, activated Notch is unequally distributed during NSC division and it has been shown to directly regulate *Egfr* transcription [110]. Next, during cytokinesis, Dyrk1a kinase is asymmetrically segregated to the EGFR^high^ daughter, where it prevents EGFR degradation [113]. Unequal EGFR expression between sibling cells appears to be a common cell-fate switch in embryonic and adult NSCs [114]. Together, this suggests that cell-intrinsic processes and signals from the niche synergistically regulate NSC fate.

EGFR and Notch are part of the signaling pathways involved in cancer and these pathways are frequently altered in brain carcinogenesis. Implications of alterations in these pathways on stem and progenitor cell division mode in the context of brain cancer will be discussed below.

### Asymmetric divisions during gliogenesis

In rodents, the generation of astrocytes starts during embryonic development and peaks at neonatal stages. Astrocytes, which are commonly identified by expression of GFAP [115], can arise from RG cells [93, 116–118], glia-restricted progenitor cells in the white and gray matters [116, 119] and from local proliferation—i.e., symmetric expansion—of differentiated astrocytes [120] (Fig. 3b). However, due to unavailability of lineage-specific markers, the sequence of developmental steps that glia-restricted precursor cells undergo before giving rise to mature astrocytes are not yet clear.

Oligodendrocyte precursor cells (OPCs), on the other hand, are characterized by the expression of platelet-derived growth factor receptor alpha (PDGFRα) [121], Olig2 [122], and NG2 [123]. They originate at different developmental stages in discrete regions of the CNS, from where they migrate before differentiating into myelinating oligodendrocytes [121, 124, 125] or protoplasmic astrocytes [119].

In the adult brain, OPCs are widespread in the gray and white matters and continue to divide throughout life, making up the largest proliferative population in the postnatal brain [126, 127]. In vivo lineage tracing of NG2^+^ OPCs has revealed that the differentiation potential of postnatal and adult OPC becomes restricted to oligodendrocytes. Live imaging of single NG2^+^ OPCs in mouse brain slices has shown that at early postnatal stages OPCs self-renew and generate differentiated progeny by undergoing either asymmetric self-renewing, symmetric proliferative or symmetric differentiating divisions [128]. Detailed immunohistochemistry analyses of dividing OPCs in the adult mouse brain have indeed confirmed the presence of symmetrically dividing OPCs producing two NG2^+^ OPCs, and asymmetrically dividing OPCs that generate one NG2^+^ OPC and one NG2-negative daughter (Fig. 3c). In vitro studies further indicate that the NG2-negative daughter cell is committed to differentiation [3]. These findings demonstrated that glial cells in the adult mammalian brain and, particularly mammalian OPCs, can undergo ACD to self-renew and generate mature oligodendrocytes at a one-to-one ratio. A first glimpse of the mechanism involved in asymmetric cell-fate determination in OPCs comes from in vitro data showing that NG2 not only tracks self-renewing fate but also instructs EGFR to co-segregate to the proliferative progeny. Thereby, each asymmetric OPC division generates one NG2^+^ OPC that activates EGFR and self-renews, and a NG2-negative cell that becomes a differentiated oligodendrocyte [3].

NG2 is a chondroitin sulfate proteoglycan (CSPG) and part of the proteoglycan family, which regulates many signaling pathways implicated in cancer, including growth factor receptor tyrosine kinases (RTKs), as well as cell–microenvironment interactions [129]. In the brain, NG2 is only expressed by OPCs and pericytes, suggesting that it constitutes a cell type-specific determinant of cell fate. Future studies are expected to reveal potential interactions of NG2 with molecules of the conserved asymmetry regulatory machinery. Such data will provide mechanistic insights into the regulation of ACD in the adult brain.

## Asymmetric cell division and cancer

Cancer is caused by the step-wise acquisition of genetic mutations, which provide a survival benefit to the affected cell(s). Despite the heterogeneous nature of the disease, most cancers can be described by a few organizing principles, the so-called “hallmarks of cancer”, a term that has been coined by Hanahan and Weinberg over the past decade [130, 131]. Among such “hallmark capabilities” of cancer cells are their ability to sustain proliferative signaling, evade growth suppressors, and resist cell death. In addition, cancer cells lose cell adhesion, change their shapes, acquire novel cell-fate characteristics and secrete factors, including metalloproteases that alter their environment. These changes are associated with the ability of cancer cells to invade adjacent tissue and to migrate to distant sites to form metastases. Moreover, it has been proposed that the acquisition of distinct hallmarks relies to a great extent on genomic instability [131].

Although it is clear that these cellular features are complementary and might indeed be mechanistically linked, individually, they provide a basic framework to understand the biological complexity of cancers. Genetic mutations linked to cancer co-opt molecular pathways that are important regulators of normal development and cellular capacities such as proliferation, differentiation, and cell-cycle and growth control. It is feasible that cancer-causing mutations also hijack ACD regulatory pathways, since these orchestrate cellular proliferation, cell-cycle progression, cell shape and fate, and possibly genomic stability. Below, we discuss how this hypothesis has been addressed in fly NBs and murine models of human cancers as well as human cancer cell lines.

### ACD as a tumor suppressor mechanism: evidence from *Drosophila* neuroblasts

**Fig. 4:**
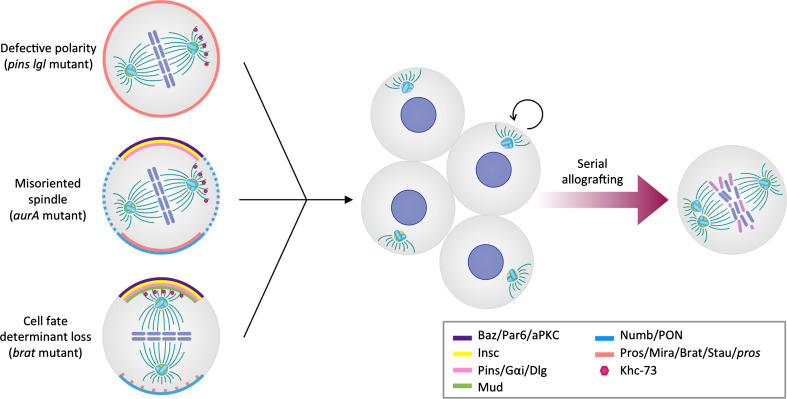
Defects in polarity, spindle orientation, or cell-fate specification disrupt asymmetric division and result in hyperproliferation and loss of differentiation of *Drosophila* neuroblasts. Serial allografting of asymmetry-defective, hyperproliferative neuroblasts leads to chromosomal abnormalities and aneuploidy

The first suggestion that loss of ACD might be involved in tumorigenesis came from discoveries in *Drosophila*. Studies of loss-of-function mutations in key regulators of ACD, including *lgl* [132], *aurA* [31, 32], *polo* [30], *numb* [10, 31, 32], and *brat* [10, 43, 44, 50], revealed hyperproliferative phenotypes in situ. In these mutants, presumably due to defective ACD, NBs divide more symmetrically and generate mis-specified progeny that fails to exit the cell cycle and differentiate, but rather proliferates continuously (Fig. 4).

To study the effects of sustained disruption of ACD in fly NBs beyond the lifespan of the fruit fly, the mutant tissues were implanted into the abdomen of adult wild-type hosts. Interestingly, the brain tissue transplants from larvae mutant for *lgl* and *dlg* [133], or for *pins*, *numb*, *mira*, *pros*, or *brat* [134] but not wild-type transplants, grew expansively and invaded the host. Upon subsequent rounds of allografting, the ACD mutant tissues contained cells with aberrant karyotypes and multiple centrosomes [134] (Fig. 4). Appearance of these defects correlated with higher ability to re-grow tumor-like masses upon re-implantation. These experiments not only support the observation that impairment of ACD and the resulting loss in cell-fate determination can cause hyperproliferation but also suggest that loss of polarity and spindle control may be an important step in the neoplastic transformation of asymmetrically dividing stem and progenitor cells [135].

The integrity of the mitotic spindle and centrosomes is paramount to a stable genome and disruption of mitotic spindle checkpoints and centrosomes have been frequently observed in human cancer. Distinct studies have investigated the relevance of centrosome integrity for normal growth. Hypermophic mutants for *polo*, *aurA*, and *dsas*-*4* fail to properly regulate the centrosome in both asymmetrically dividing NBs and symmetrically dividing epidermal cells. Surprisingly, allografting experiments have shown that while transplants of larval brain tissues from *polo*, *aurA*, or *dsas*-*4* mutants lead to formation of masses reminiscent of tumors, implanted epidermal tissue from the same mutants never forms masses [136]. These observations indicate that loss of centrosomal integrity can cause hyperplastic growth of asymmetrically dividing cells, but not of cells undergoing symmetric divisions. Overexpression of the protein kinase Sak/Plk4 in NBs leads to supernumerary centrosomes, aberrant mitotic spindle positioning, and mild hyperproliferation. Upon allografting, NBs overexpressing Sak also form tumor-like masses, although at lower rates than ACD mutant NBs [137]. Given that only a small fraction of the Sak mutant cells display mislocalized expression of cortical regulators and therefore impaired ACD [137], the data suggest that losing centrosomal integrity in itself does not initiate hyperproliferative phenotypes and associated mass formation. Instead, these seem to be secondary effects of ACD disruption.

Due to the intertwined control of polarity, spindle orientation, cell-fate determination, and proliferation, it is challenging to uncouple those events and to reveal a temporal sequence of defects. However, mounting genetic evidence suggests the provocative possibility that failure of basal cell fate determinants to segregate properly initiates a cascade of events that starts with aberrant proliferation of NBs and that ultimately leads to the neoplastic transformation of stem or progenitor cells. Hyperproliferative NBs acquire spindle defects such as supernumerary centrosomes and overrule spindle checkpoints, which causes genomic instability and thus introduces cellular phenotypes similar to those found in human brain cancer [138]. Large-scale forward genetic screens in *Drosophila* cancer models will be useful in clarifying the sequence of events leading to massive outgrowth of larval brain tissue and those data are expected to inform studies of human brain cancer development.

### Relevance of ACD during cancer formation

#### Genetics of ACD regulators in cancer

Cancer is essentially a genetic disease and arises from a cell that has acquired a cancer-initiating mutation. The accumulation of additional genetic and/or epigenetic alterations cause the cancer cells to further progress and grow, invade, and metastasize. Most frequently, mutations are single-nucleotide changes or point mutations. Additional genetic aberrations found in cancer cells are smaller chromosomal changes such as translocations and insertions, as well as regional amplifications and deletions. However, many cancer cells also exhibit loss or gain of entire chromosomes [139]. Emerging evidence indicates that key regulators of ACD are mutated in multiple cancer types and introduction of such mutations in murine models cause cancer-associated phenotypes (Table 1). For instance, a recent genome-wide screen for micro-deletions across a range of primary and cultured human tumor cells identified the deletion of polarity regulators in numerous human epithelial tumor cells. Such deletions include *PARD6* (PAR6) in lung cancer, *PARD3* (PAR3) in lung, head and neck, esophagus, prostate and bladder cancers, and *DLG2* in lung and cervical cancers [140, 141].

**Table 1:** Asymmetric cell division regulators and their role during cancer formation and progression

Gene	Chromosomal location	Genetic alteration and/or expression in human tumors	Cancer-associated phenotypes in in vivo mouse models
*AURAK*	20q13.2–q13.3	Amplified and overexpressed in diverse human tumors [194], including breast [195], ovarian [196], gastric [197], bladder [198], and pancreatic [199] cancers	Pharmacological inhibition in breast [200] and ovarian [201] cancer cells and siRNA-mediated depletion in laryngeal cancer cells [202] prevent metastasis upon xenotransplantation
*CSPG4* (*NG2*)	15q24.2	Overexpressed in astrocytomas [129] and oligodendrogliomas [3]	Pericyte deficiencies in *Cspg4* knockout mice lead to aberrant tumor vascularization [203]
*DLG2*	11q14.1	Localizes within a large common fragile site [204]. Expression downregulated in oligodendroglioma [3]	Not determined
*HUGL*-*1*	17p11.2	Gene loss in 75 % of colorectal cancers [205]. Reduced expression in melanoma, breast, lung, prostate [206], and ovarian cancers [207]	*Lgl1* knockout mice have pre-cancerous rosette-like structures in the brain. Neural progenitor cells fail to differentiate and to exit the cell cycle [147]
*HUGL*-*2*	17q24–q25	Loss or aberrantly localized in gastric adenocarcinomas [208]. Reduced expression in colorectal and breast cancers [209]	Forced expression in breast cancer cell lines reduces their metastatic potential upon subcutaneous xenotransplantation [210]
*MSI1*	12q24.1–q24.31	Upregulated in oligodendrogliomas [3], astrocytomas [211], colorectal tumors [212], and endometrial cancers [213]	*Msi1* knockdown reduces growth of xenografted glioblastoma [214] and colon adenocarcinoma cells [215]
*MSI2*	12q24.1–q24.31	Increased expression in CML [153]	Loss of function in HSCs expressing NUP98-HOXA9 reduces leukemia growth in vivo [153]
*NUMB*	14q24.3	Reduced protein levels in some mammary carcinomas [151], NSCLC [216] and blast crisis of CML [153]	Ectopic expression in HSCs transduced with BCR-ABL and NUP98-HOXA9 reduces the incidence and propagation of blast crisis in vivo [153]
*PARD3*	10p11.21	Reduced expression in melanoma, breast, lung, and bladder cancers [149]	In the presence of relevant oncogenic mutations, loss of *Par3* favors formation of keratoacanthomas [150] and increases mammary tumor growth and metastasis [149]
*PARD6A*	16q22.1	Increased expression [217] or mis-localized [173] in low- and high-grade breast cancers	Expression of a dominant negative form in mammary carcinoma cells reduces incidence of metastases [166]
*PLK1*	16p12.2	Increased expression in several tumors, including NSCLC [218], gastric [219], ovarian [220], prostate [221], bladder [222], breast [223], head and neck [224] cancers, and gliomas [225]	siRNA-mediated depletion in bladder cancer [222] and GBM [226] cell lines and pharmacological inhibition in breast cancer [223] and glioma cells [226] impairs/delays tumor growth in xenograft models
*PRKCI*	2p21	Overexpressed in NSCLC [227], breast [228], ovarian [229], and prostate [230] cancers	Expression of a dominant negative form in lung cancer cells [231] and siRNA-mediated depletion in prostate cancer cells [230] reduce tumorigenicity in vivo. Required for Ras-mediated transformation of intestinal epithelial cells [168]
*SCRIB1*	8q24.3	Reduced expression or mis-localized in glioma [232]	Loss of *Scrib* predisposes to cMyc transformation in mammary epithelia [233]
*TRIM3*	11p15.5	Loss of heterozygosity in some gliomas [234]. Downregulated in a subset of gliomas [235], including oligodendrogliomas [3]	Knockdown increases incidence of PDGF-driven gliomas in *p21*-deficient mice [235]

Malignant brain tumors are classically categorized into three main variants: astrocytomas, oligodendrogliomas, and mixed oligoastrocytomas. Grade IV astrocytoma, also known as glioblastoma multiforme (GBM), is the most aggressive type of glioma. The Cancer Genome Atlas has provided a comprehensive view of the genomic landscape of GBM [142]. The study revealed that a small number of signaling pathways are frequently mutated in these tumors. Single-nucleotide variations within genes implicated in ACD regulation are rarely found in GBM (Andor and Petritsch; unpublished observations). Yet, expression of ACD regulators is frequently altered in many types of human cancers (see Table 1 for a list), including gliomas [3]. It is therefore likely that regulators of ACD are subjected to epigenetic and/or posttranslational modifications that influence their expression levels in cancer cells. Examples of such regulations have been provided for a small number of ACD regulators and these will be discussed below.

#### ACD and the cellular origin of (brain) cancer

Most cancers arise from a single cell, i.e., they have a clonal origin. Genetically engineered mouse model (GEMM) studies suggest that in several organs, including the brain, the cellular origin of cancer might be a stem or progenitor cell.

Despite their clonal origin, many cancers are diverse and have significant intratumoral genetic and phenotypic heterogeneity. Underlying genetic factors and the unique microenvironment within each tumor contribute to its individual evolution and heterogeneity. The effects of genetic heterogeneity and the microenvironment on the mode of cell division of the stem and progenitor cells of origin are yet unknown. Our recent studies indicate that the rates of ACD are different between tumors of a single tumor type [3] and may have to be determined individually for each tumor.

Given the difficulty of studying cancer initiation in human patients, the development of GEMMs that recapitulate key aspects of the disease has been crucial for elucidating the cellular and molecular events occurring at different stages of tumorigenesis. Sophisticated GEMMs modeling key genetic alterations in the core signaling pathways found to be mutated in GBM, namely the p53 pathway, the Retinoblastoma (RB) pathway and the RTK signaling pathways, including deletion of the neurofibromatosis type 1 (NF1) gene [143], have recently become available [144]. These mice have been used to investigate the cellular origin of astrocytoma and oligodendroglioma.

These GEMM studies have shown that the sequence by which cellular controls become dysregulated during oncogenesis can vary between cancer types. However, increased proliferation, enhanced self-renewal, and evasion from cell cycle control tend to be early events during cancer development. In contrast, immortalization, genomic instability, invasion and metastasis are considered intermediate or late events [131]. The molecular mechanisms and temporal regulation of defects in polarity and ACD in human cancer initiation and progression remain poorly understood. Below, we discuss the emerging role of ACD dysregulation in promoting hallmark capabilities of cancer cells with a focus on data obtained from GEMMs of glioma.

#### Abnormal proliferation and self-renewal

Asymmetric divisions are a key mechanism to ensure tissue homeostasis. In normal stem and progenitor cells, ACD balances proliferation and self-renewal with cell-cycle exit and differentiation. Disruption of ACD leads to aberrant self-renewal and impairs differentiation, and could therefore constitute an early step in the neoplastic transformation of stem and progenitor cells.

**Fig. 5:**
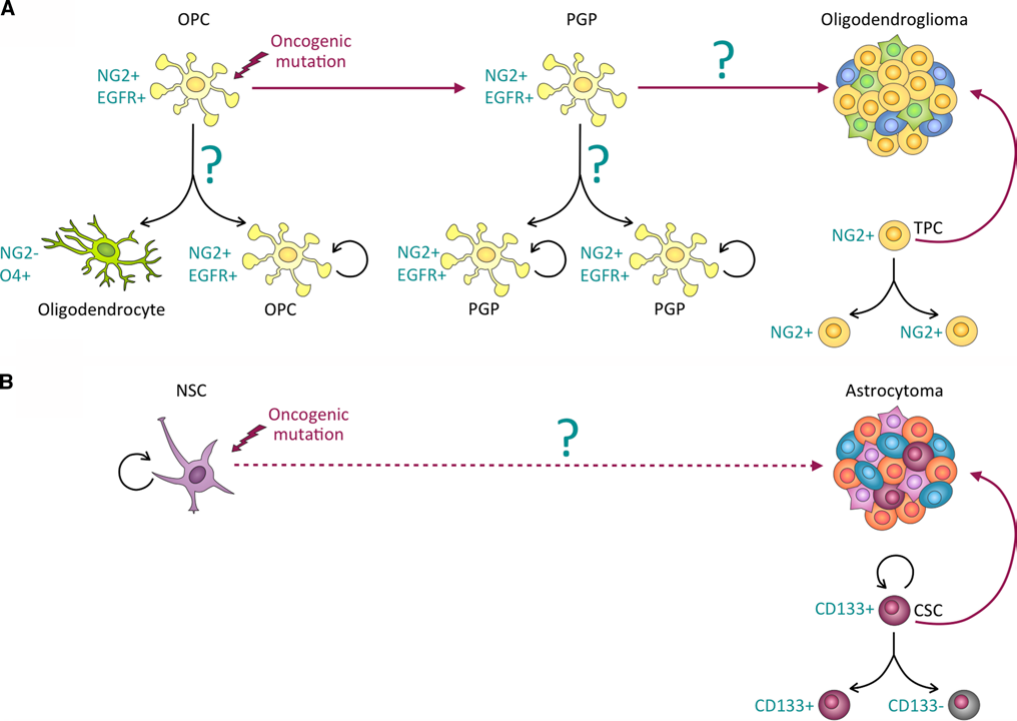
Cellular origin of malignant gliomas. **a** Oligodendrocyte precursor cells (*OPCs*) in the adult brain can divide asymmetrically to give rise to a self-renewing NG2^+^ OPC and a differentiated NG2^-^O4^+^ oligodendrocyte. In *p53* hemizygous mice, expression of the viral oncogene *verbB* in OPCs disrupts asymmetric OPC division and causes hyperproliferation of symmetrically dividing premalignant glioma precursor (*PGP*) cells, which, following unknown transformative events, eventually give rise to oligodendrogliomas. Similar to their human counterparts, murine oligodendrogliomas contain symmetrically dividing NG2^+^ tumor-propagating cells (*TPCs*). **b** Inducing oncogenic mutations of core signaling pathways (e.g., inactivation of the p53 and Rb tumor suppressor pathways and activation of RTK signaling) in mouse neural stem cells (*NSCs*) leads to formation of tumors with features of high-grade astrocytomas. CD133^+^ cancer stem cells (CSCs) isolated from human high-grade astrocytomas self-renew through symmetric and asymmetric cell divisions

In a GEMM of oligodendroglioma, in which the viral oncogene *verbB* is expressed under the control of the *S100β* promoter in a *p53* hemizygous background [145], NG2^+^ OPCs expand and initiate tumor development [146]. Detailed cellular analyses in this model demonstrated that premalignant NG2^+^ OPCs display a higher frequency of symmetric self-renewing divisions at the expense of asymmetric differentiating divisions, when compared with non-neoplastic OPCs (Fig. 5). Remarkably, NG2^+^ OPC-like cells isolated from human oligodendrogliomas also exhibit defective ACD, leading to an overproduction of daughter cells that inherit both NG2 and EGFR [3]. Furthermore, bioinformatic analyses revealed altered expression of 15 conserved ACD regulators in human oligodendroglioma [3]. This suggests that defects in ACD may occur during neoplastic transformation of OPCs and may contribute to oligodendroglioma formation. An emerging scenario for the early events of OPC transformation is that NG2 levels are upregulated early in tumorigenesis in response to expression of verbB, which mimics constitutively active EGFR signaling. Abundance of both molecules disrupts the asymmetric distribution of NG2 and EGFR during OPC division. In the normal adult brain, the OPC progeny negative for these two molecules enters the path of differentiation. In premalignant OPCs, however, NG2/EGFR segregates to both daughter cells, causing their abnormal proliferation (Fig. 5). The molecular details of how NG2/EGFR asymmetry is established in OPCs remain to be fully elucidated, and it is possible that verbB expression also affects other conserved ACD regulators.

Studies of the central nervous system of mice lacking *Lgl1*, one of two mammalian orthologues of *Drosophila* *lgl*, have revealed precancerous brain lesions and prenatal death due to hydrocephalus. Misregulation of Lgl1 disrupts downstream localization of cell-fate determinants, decreases differentiation, and increases proliferation and apoptosis. Furthermore, neural progenitors in prenatal *Lgl1*-knockout mice show loss of AJs [147]. It is interesting that loss of polarity or cell-fate regulation through mutation of *apkc*, *lgl*, or other polarity regulators is sufficient to induce massive hyper-proliferation in *Drosophila* NBs. In contrast, in mammalian epithelia and progenitor cells, loss of ACD regulators leads to tissue disorganization and hyperplasia [147, 148], but does not appear to be sufficient to initiate tumor formation [149, 150]. Functional redundancies between the regulators of ACD have been observed in *Drosophila* and they may mask the role of ACD in tumor initiation in mammalian GEMMs.

#### Growth suppressor evasion

Mounting evidence points to a role for the cell-fate determinant NUMB as a tumor suppressor in distinct types of human cancers. In breast cancers, NUMB protein levels are frequently reduced or lost and inversely correlate with tumor grade [151] and patient prognosis [152]. In primary breast tumor cell cultures, those with low or no NUMB expression, referred to as class 1, not only display increased NOTCH activity [151] but also a reduction of TP53 protein levels [152]. Forced-expression of *NUMB* in class 1 cells significantly reduces NOTCH activity and cell proliferation [151] and restores normal TP53 levels [152].

Numb has been found to physically interact and stabilize p53 by preventing its ubiquitination and degradation induced by the E3 ubiquitin ligase HDM2 [152]. In primary *ErbB2*-driven mouse mammary carcinoma cells, p53 is unstable, and attenuated p53 levels switch the mode of cell division from asymmetric to symmetric, leading to geometric cell expansion [5]. Although it is not clear whether in this model the levels of Numb protein are also affected, these observations suggest that downregulation of NUMB/TP53 in human breast cancers may cause a bias towards symmetric divisions.

Reduced NUMB levels are also observed during the progression of human chronic myeloid leukemia (CML) to blast crisis and, again, this is associated with increased NOTCH signaling and reduced TP53 activity [153]. In a mouse model of myeloid blast crisis driven by co-expression of the oncogenic fusion proteins BCR-ABL and NUP98-HOXA9, it was found that overexpressing Numb or inhibiting Notch activity reduces the incidence and propagation of blast crisis. Importantly, in the absence of p53, ectopic Numb had no impact on leukemic cell growth, indicating that the effects of Numb are solely dependent on p53.

In normal mouse hematopoietic stem cells (HSCs), Notch activity is required for the maintenance of an undifferentiated state [154]. When isolated and cultured under differentiating conditions, HSCs divide to give rise to a Notch^+^ HSC and a differentiating daughter that inherits Numb and downregulates Notch. Transduction with *NUP98*-*HOXA9* causes Notch^+^ HSCs to grow expansively [4]. This suggests that Notch overactivation may mediate leukemic growth, at least in part, by promoting symmetric expansion of more immature cells.

Underexpression of NUMB in the above-mentioned tumors appears to be a consequence of posttranscriptional modulation and not of genetic alterations affecting the *NUMB* locus. In breast cancer, loss of NUMB expression results from increased proteasomal degradation [151]. Downregulation of Numb during blast crisis has been proposed to be mediated, at least in part, by Musashi2 (Msi2) [153]. Depletion of MSI2 in cell lines from patients with CML blast crisis increased NUMB protein levels and led to reduced cell proliferation and apoptosis induction in vitro [155]. Similarly, *Msi2* knockdown in mouse HSCs transduced with *BCR*-*ABL *and *NUP98*-*HOXA9* resulted in significant leukemic growth impairment in vivo [153]. The Msi family members are evolutionary conserved RNA-binding proteins. In *Drosophila*, Msi is required for ACD of the sensory organ precursor cell in the peripheral nervous system and its main in vivo target is *tramtrack69* mRNA [156]. Two Msi family genes have been identified in mammals, *Msi1* and *Msi2*. Msi1 is selectively expressed in neural progenitor cells [157] where it has been shown to repress *Numb* translation [158]. It is thus thought that Msi2 may regulate Numb protein levels by a similar mechanism.

The observation that NUMB stabilizes TP53 provides a connection between evasion of growth suppression and genomic instability. Disruption of ACD and loss of p53 due to lack of NUMB are therefore expected to increase the possibilities of malignant transformation. Moreover, by eliminating the antagonistic effect on NOTCH signaling, reduced NUMB levels would also lead to maintenance of an undifferentiated state and abnormal self-renewal. Further studies are required to test this model and to determine if the mechanisms operating in breast cancer and the blast crisis of CML are conserved in tumors from other tissues. Loss of p53 in the adult mouse brain expands the pool of type A cells and quiescent B cells and leads to increased self-renewal, but not to tumor formation [159]. In a GEMM of astrocytoma, combined loss of *p53* and *Pten* impairs NSC differentiation and induces tumor formation [160]. Ectopic expression of a mutant form of p53 in adult GFAP^+^ cells suggests that, although NSCs are initially accumulating mutations, progenitors expressing Olig2, a marker of OPCs and TAPs, are actually expanding and forming tumors [161]. It will be interesting to determine how Numb and other ACD regulators may impact gliomagenesis through evasion of cell cycle control.

#### Invasion and metastasis

**Fig. 6:**
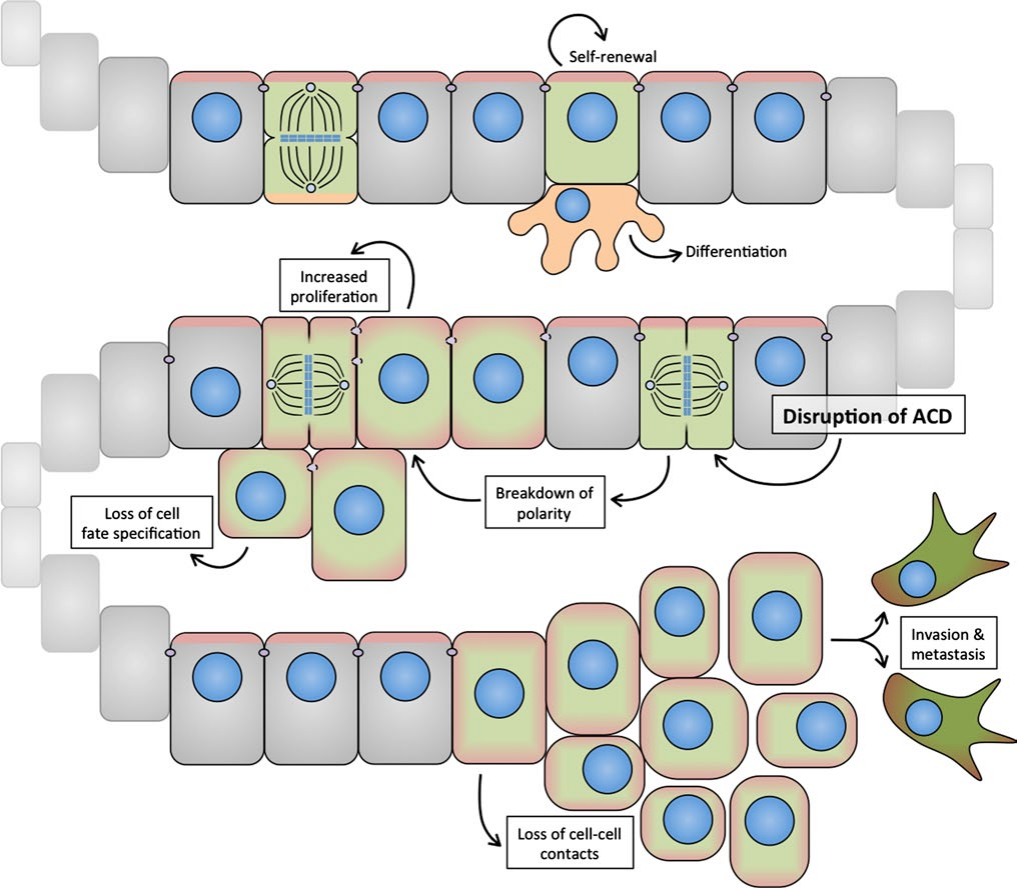
Loss of polarity, ACD, and tissue architecture during EMT may contribute to invasion and metastasis. Apical (self-renewal, *red*) and basal (pro-differentiation, *orange*) signals are segregated to opposite compartments in asymmetric divisions of some epithelial progenitor cells (*green*). Disruption of ACD may lead to breakdown of polarity and increased inheritance of pro-proliferative signals usually confined to the apical domain, and a concomitant loss of cell-fate specification. Disruption of apical domain and AJ stability contribute to loss of epithelial integrity, and may be important steps in tumor cell invasion and metastasis

The molecular mechanisms leading to loss of polarity are still only partially understood. However, recent work has revealed that many important oncogenes act as key (mis)regulators of polarity. Given the frequency at which polarity defects are observed in human cancers, particularly in high-grade tumors, loss of polarity may be a necessary step during tumor progression. Epithelial mesenchymal transition (EMT) is thought to be an important source of invading and metastasizing cells (Fig. 6) [162]. Strikingly, recent work has shown that maintaining aPKC signaling at the apical surface is sufficient to prevent EMT in non-small cell lung carcinoma (NSCLC) cells in vitro [163].

#### TGF-β and polarity

Transforming growth factor beta (TGF-β) signaling has long been implicated in EMT [164] and is frequently upregulated in tumor cells [165]. Recent studies have shown that TGF-β-mediated transformation involves direct disruption of epithelial cell polarity that precedes breakdown of AJs and epithelial organization. TGF-β binding to the TGF-β receptor III leads to direct phosphorylation of Par6 [166], via a partially aPKC-dependent mechanism [163]. Phosphorylation of Par6 leads to the breakdown of the Par complex, subsequent loss of apical G-protein activity, and breakdown of AJs [163, 167]. Hypothetically, loss of polarity in epithelial cells could switch the plane of division from a perpendicular to a planar orientation. In a planar division, the apical domain is bisected and the cell divides symmetrically. In contrast, in a perpendicular division, the basal cell may no longer be bound to the epithelium and can acquire distinct fate and migrate further basally. Whether this switch of division occurs and is involved in EMT remains to be tested (Fig. 6).

#### RTK signaling and polarity

Dysregulation of the EGFR family of RTKs is very common in many cancers, including astrocytoma and oligodendroglioma. In epithelial tissues, EGFR family members have been implicated in regulation of polarity, which is an essential component of ACD. Activation of ErbB2 [168] or EGFR [169], both EGFR family members, leads to increased activity of mitogen-activated protein kinase (MAPK) family members MEK and ERK via increased Ras activity. ErbB2-mediated transformation of mammary epithelial cells directly disrupts the Par3/Par6/aPKC complex and this disruption leads to a breakdown of cellular polarity and epithelial structure [170]. It appears that, at least in the case of ErbB2-mediated transformation, polarity defects do not arise secondary to increases in proliferation, but rather polarity pathways are directly targeted by the oncogenic transformation during very early stages of tumor formation.

Moreover, the archetypal tumor suppressor PTEN, which is a negative regulator of RTK signaling, has also been implicated in the maintenance of epithelial polarity. Polarized localization of PTEN to the apical membrane dephosphorylates phosphatidylinositol 3,4,5-trisphosphate (PIP3), antagonizing phosphatidylinositol 3-kinase (PI3K) signaling at the apical domain of the cell. This restriction of PI3K activity at the apical surface appears to play a key role in establishing polarity, as localization of PTEN to the baso-lateral membrane results in the recruitment of apical protein Cdc42 to the lateral wall, while ablation of PTEN activity disrupts apical polarity [171].

#### Polarity complex and adherens junction breakdown

There is an intimate relationship between polarity and maintenance of AJs. Indeed, loss of Par3, Par6, or Cdc42 individually destabilize tight junctions, leading to disruption of epithelial polarity [172–175]. Studies of polarity in tumor initiation have suggested that disruption of normal epithelial organization may have cell type-specific, possibly opposite, effects on tumor initiation and progression. Loss of Par3 disrupts polarity is skin epithelia, and inhibits the initiation and progression of low-grade papilloma while promoting the development of high-grade keratoacanthomas [150]. Increased invasion following loss of Par3 may result from the induction of aPKC-dependent activation of JAK/STAT signaling, which induces metallopeptidase 9 (MMP9) expression by transformed mammary epithelial cells [149]. In a transplantation model of mammary carcinoma, loss of Par3 cooperates with ErbB2 to destabilize E-cadherin junctions and aberrantly activate Tiam1-Rac-GTP signaling. Par3 loss also induces invasive behavior and metastases formation in this model. Loss of Par3, however, does not alter the weight of the primary tumor and fails to induce molecular changes associated with EMT [172]. Thus, loss of polarity may increase invasiveness by upregulating expression of extracellular matrix degrading enzymes as well as by disrupting inhibitory cell–cell contacts.

The temporal regulation of polarity defects and how they arise to impact tumor initiation and progression in human cancer is still unclear. In particular, the causal relationship between disruption of ACD and associated loss of cellular polarity is not yet known.

### Clinical relevance of ACD in tumor-propagating cells

Glioblastoma multiforme (GBM) are fast-growing grade IV astrocytomas and extremely resistant to radiation- and chemotherapy. Oligodendrogliomas, on the other hand, are slow-growing cancers that are very responsive to chemotherapy [3, 146]. Yet, both tumors can arise from immature neural progenitors. These wide-ranging clinical manifestations in tumors with relative similar cellular origin provide an interesting conundrum. GEMM studies have provided evidence that astrocytomas originate from NSCs and OPCs [176, 177], whereas oligodendrogliomas originate from OPCs [146] (Fig. 5). OPCs are more sensitive than NSC to the growth inhibitory effects of temozolomide, an alkylating agent that is used as standard treatment for high-grade gliomas [146]. It is therefore speculated that the cellular origin of the two types of glioma (i.e., NSCs in GBM and OPCs in oligodendroglioma) in part determines their distinct therapy responses [178].

At least two models attempt to explain how a single cell of origin can lead to a heterogeneous tumor, displaying expression of markers of distinct brain cell types at various stages of differentiation. The stochastic model has long been established and claims that cancer cells acquire mutations stochastically and those mutations that provide a survival benefit will expand the affected cell population. In this model, cancer evolves from a single mutated cell (clone), through clonal expansions. On the other hand, the cancer stem cell (CSC) model postulates that only some cancer cells, namely those with stem cell properties, are tumorigenic. These CSCs are proposed to generate cellular hierarchies similar to NSCs by self-renewing and giving rise to progeny that then “differentiates” into heterogeneous tumor cells. Recently, an extended model of cancer development has been provided, which combines the stochastic and the CSC hypothesis by claiming that different cancer cell populations may evolve and these interact with each other and with the cancer microenvironment, thereby promoting cancer growth and development of therapy-resistant subpopulations [179]. Several studies have indeed provided evidence that diverse human cancers contain cellular subpopulations that exhibit stem-like features, such as multi-potentiality and self-renewal ability. However, in contrast to their non-neoplastic counterparts, in CSCs the self-renewal machinery is dysregulated, leading to uncontrolled growth and heightened malignant potential when compared to the bulk of tumor cells [180]. Due to their ability to re-grow tumors with parental phenotypes when xenotransplantated into immunocompromised mice, these cells are also often called “tumor-propagating cells” (TPCs), tumor-initiating cells, or stem-like cancer cells. TPCs include the classically defined stem-like CSCs, as well as those tumor cells with heightened malignant capacity that share more attributes with lineage-restricted progenitor cells rather than stem cells.

CSCs are common in anaplastic astrocytoma and GBM [181–183] and are present in some anaplastic, high-grade oligodendrogliomas [184]. CSCs have been associated with therapy resistance to radiation [185, 186] and conventional chemotherapy [187, 188]. This has led to the speculation that while the majority of tumor cells are eliminated by conventional treatment, the CSCs survive to re-grow the tumor and are thus culprits for tumor recurrence. This model has been supported by a sophisticated study in a murine glioblastoma model, which showed that a relative quiescent Nestin^+^ neural progenitor population survives treatment with the alkylating agent temozolomide, while the tumor bulk shrinks. Moreover, the Nestin^+^ population significantly contributes to tumor re-growth [189].

The resemblance to stem and progenitor cells by CSCs and some TPCs, respectively, has raised the question of whether they undergo ACDs. CD133 is a surface marker for CSCs in GBM [182] and cultured CD133^+^ CSCs indeed asymmetrically segregate CD133 during mitosis [6]. Molecular analyses have recently revealed that CD133 regulates CSC maintenance by activating the PI3K/Akt signaling pathway, which is frequently upregulated in glioma. CD133 physically interacts with the PI3K regulatory subunit p85 and thereby activates Akt signaling, promoting CSC self-renewal and tumor-initiating potential [190]. The cell-fate determinant Numb has been reported to asymmetrically segregate to CD133^high^-expressing GBM cells and to specify their stem cell fate [191]. Interestingly, when oligodendroglioma cells were fractionated into stem cell-like and progenitor-like subpopulations, the progenitor-like subpopulation, characterized by high expression of the OPC marker NG2, but not the stem cell-like population, was capable of initiating tumor growth upon orthotopic implantation [146]. This indicates that oligodendroglioma harbor a population with progenitor-like features, rather than stem cell-features. We therefore call these cells TPCs rather than CSCs. It is feasible that a progenitor-like TPC has limited self-renewal potential and may eventually differentiate, which may explain the slower tumor growth of oligodendroglioma. Examination of NG2^+^ TPCs isolated from human surgical glioma specimens from patients prior to treatment revealed that these cells divide predominantly symmetrically [3]. Thus, asymmetry-defective oligodendroglioma TPCs presumably expand the tumor by non-hierarchical, symmetric divisions.

A major open question with regards to glioma CSCs and TPCs and in relation to cell division modes is about their predecessor in humans. Do CSCs have a common predecessor, such as a NSC, and do TPCs arise from progenitor cells instead? Such a direct lineage relationship of the CSCs and TPCs and their non-neoplastic counterpart is suggested by their biological similarities. Indeed, studies with GEMMs of glioma have suggested that NSCs can act as cellular origin of astrocytoma [176, 192]. Alternatively, CSCs may arise de novo by de-differentiation of, for example, OPC-like cells, which is further supported by the observation that high-grade astrocytoma can arise from NG2^+^ OPCs [177] and even from mature astrocytes [193]. These studies suggest that depending on cellular context and the nature of the mutations, several cell types have the capacity to give rise to CSCs and TPCs. Given that TPCs can divide asymmetrically [6], but astrocytes for example divide mostly symmetrically [120], these data indirectly suggest that a subset of tumor cells may acquire asymmetric division modes.

In summary, in glioma, the capacity for tumor propagation is not restricted to NSC-like cells, but can also be kept by progenitor-like cells, which are more accurately referred to as TPCs. Regardless of how these distinct phenotypes are acquired, it is clear that tumors are comprised of multiple cellular populations that may not be equally impacted by conventional therapies. Understanding why some populations of cells acquire and/or retain ACD, while others expand symmetrically, may prove useful in understanding why distinct cancer cell types are unequally affected by treatment.

## Concluding remarks

Groundbreaking work in *Drosophila* has helped to uncover many of the molecular mechanisms and cellular processes underlying ACD regulation in mammalian cells. Recent questions have begun to push the limits of invertebrate model systems, in particular with respect to the great variety of progenitor cell types and ACD mechanisms present in mammals.

As evidence mounts for adult progenitor cells as a likely cellular origin of distinct human cancers, the importance of understanding the molecular mechanisms underlying ACD regulation in different types of progenitor cells become more urgent. In-depth cellular analyses of complex and difficult-to-treat tumors, such as high-grade astrocytoma, constantly reveal new types of subpopulations that may be resistant to traditional therapy. New research will allow therapeutics to target defects in ACD, and associated changes in polarity, spindle orientation, and cell-fate decision, in different types of cancer cells.
